# Early excellence and future performance advantage

**DOI:** 10.1371/journal.pone.0306134

**Published:** 2024-06-25

**Authors:** Tiantian Wang, Yaping Zhong, Xin Wei

**Affiliations:** 1 School of Physical Education, Hubei University of Technology, Wuhan, Hubei, China; 2 Sports Big-data Research Center, Wuhan Sports University, Wuhan, Hubei, China; Universiti Malaysia Terengganu, MALAYSIA

## Abstract

**Objectives:**

The objective of this study was to examine the impact of athletes achieving excellence at different ages (excellent age) on their subsequent performance development. The aim was to deepen understanding of the interplay among talent, training, and athletes’ performance development. Additionally, the study aimed to provide insights for athletics coaches to better identify talent and devise more effective personalized long-term training plans.

**Design:**

This was a cross-sectional study.

**Method:**

A hierarchical linear model was employed to analyze the correlation between excellent age and subsequent performance development in a cohort of 775 elite track and field athletes. This analysis was expanded upon by the application of a general linear regression model, which was used to explore the relationship between excellent age and peak age, peak performance, as well as the growth in performance during adulthood.

**Results:**

As athletes reached excellence at later ages, their peak performance exhibited a U-shaped pattern(p <0.001), initially decreasing and then rising. Simultaneously, their peak age became increasingly advanced(p <0.001), with a progressively larger performance improvement during adulthood(p <0.001). In various disciplines, excellent age is negatively correlated with peak performance for speed athletes(p = 0.025), exhibiting a U-shaped pattern for endurance athletes(p = 0.024), and showing no significant correlation for fast-power athletes(p = 0.916).

**Conclusions:**

Athletes who achieve excellence either early or later often show more remarkable future developments. However, there are significant distinctions in the age at which these athletes reach their peak performance and the pace of improvement leading up to it. Those who excel early may possess greater innate athletic talent, whereas those who excel later may exhibit superior training adaptability. Consequently, an athlete’s early performance can predict his/her future performance trajectory, offering support for individualized long-term training plans. In summary, the age at which athletes achieve excellence may bring different advantages to their future athletic performance and development. This implies that we should harness these differences to uncover each athlete’s maximum potential.

## 1. Introduction

In the current context of intense global sports competition, many national sports organizations have started to invest resources in a more efficient manner by identifying potential athletes to achieve better results [1, 2]. Talent identification programs aim to identify athletes with high potential for success in senior elite sports [3]. However, due to the multitude of factors affecting the development of athletic talent, the accuracy of, talent identification has remained consistently low [4]. It has been suggested that the main factor is genetic endowment but also the capacity to improve with training [5]. Most coaches believe that differences in talent (genetic endowment) determine who will succeed, [6] supporters of the Theory of Deliberate Practice emphasize that sports performance is the result of athletes’ focused, effortful training [7, 8]. Bailey, Morley [9] introduced the Differentiated Model of Giftedness and Talent, which states that an athlete’s performance development is shaped by the joint influence of talent (innate attributes individuals possess within a given domain) and aptitude (the ability acquired through deliberate practice within that domain).

Scholars have endeavored to elucidate the influence of innate talent and subsequent training on the performance development of athletes through a multitude of empirical studies [10]. Some studies have compared the training effects of identical twins to ascertain the extent to which genetic factors influence various athletic abilities. Other studies have utilized direct measurement of changes in athletes’ physical attributes after a training period to evaluate their trainability [11]. Nevertheless, the majority of these studies have concentrated on physical fitness or body morphology, which has restricted their applicability to talent identification and development in athletes. Additional research endeavors to identify early indicators of talent development from the perspective of long-term athlete development and talent identification, such as athletic performance, physical attributes, and even genetics [12, 13]. Currently, the prevailing approach to assessing athletes’ future development is to evaluate their performance in age-related competitions [4]. Research has shown that the predictive accuracy of talent identification significantly improves from the ages of 16 to 17 [14–16]. A further study has demonstrated that the performance progression from lower to higher stages is a critical indicator for successful prediction [17]. Furthermore, it has been established that performance improvement in the five years preceding peak age is a key factor in determining whether an individual can achieve higher peak performance [18]. These studies indicate that by elucidating the relationship between talent, training, and performance to some extent, the identification of talent can be effectively improved, thus further enriching our comprehension of athletes’ long-term competitive development.

The age at which athletes attain an excellent level of performance partly reflects the combined impact of their innate talent and the training they undergo [19]. Therefore, we propose the hypothesis that as athletes reach different ages of excellence, significant differences will emerge in their subsequent performance development. An investigation of the relationship between these two factors will contribute to a deeper understanding of the interplay among talent, training, and athletes’ performance progression. Furthermore, this study will provide athletics coaches with insights into the age characteristics of athletes’ competitive development, aiding them in better talent identification and the formulation of more effective personalized long-term training plans.

## 2. Method

### 2.1. Variable

1. **Excellence**: the ability of an athlete to compete in IAAF events at the F level or above and have a score of –0.6σ (standardized score) in the competition (the mean performance of athletes was –0.6σ when they were 20 years old in this study);
2. **Excellent age:** the age when the athlete’s annual best performance first reaches Excellence;
3. **Peak age:** the age that corresponds to the apex of the athlete’s performance trajectory (performance trajectory: an inverted U-shaped curve fitted by a hierarchical linear model);
4. **Peak performance:** the performance that corresponds to the apex of the athlete’s performance trajectory;
5. **Performance increases during the adult stage (PIA):** the difference between an athlete’s peak performance and their performance at the age of 18.

### 2.2 Data collection and processing

Athletes who had participated for the first time in the former International Amateur Athletic Federation (IAAF), now called World Athletics, from 1993 to 2007 and had won at least one of the top eight positions in track and field events at the World Championships or Olympics were selected for the study (Because the study found that the standard error of the model results increased rapidly after 2008). To avoid the effect of equipment divergence, the research solely focuses on specific events, including the 100 m, 200 m, 400 m, 800 m, 1 500 m, 5 000 m, 10 000 m, as well as the pole vault, high jump, long jump, and triple jump.

All data were collected from the statistics section of the IAAF website (https://www.iaaf.org/home). The IAAF publishes annual top lists categorized by athletic discipline and gender. The data for this study consists of athletes’ competition records from 1993 to 2020. The criteria for including or excluding these data are as follows: (1) To ensure equal competition regulation standards across age categories, we only included outdoor results. (2) Results obtained with illegal wind speeds (≥ 2.0 m/s) and sprint results without electronic timing were excluded. (3) Athletes with missing information, such as date of birth and previous year’s performance, were excluded. Finally, 857 athletes met the criteria of this study, for a total of 227,239 entries.

The data from a total of 227,239 were preprocessed as follows: (1) A Z-score normalization of athletes’ performance by events and gender was performed; (2) any standard deviation of performance less than −4.5 was deemed an outlier and eliminated; (3) only the best annual performance of the athlete’s participation information was retained; (4) a preliminary model was established; (5) samples with the model opening upwards were removed; and (6) samples with peak ages outside the individual age range were also removed. Finally, 775 athletes with 14,317 performances were used in this study. As the data were based on publicly available resources, no informed consent was obtained.

### 2.3 Statistical model

#### 2.3.1 Hierarchical linear model

Based on previous research, [18, 20] a hierarchical linear model was used in this study.


$$Y_{\text{ti}}=\pi_{0i}+\pi_{1i}\;\text{(}a_{ti}\text{-L)}+\pi_{2i}\;{\text{(}a_{ti}\text{-L)}}^{2}+e_{ti}$$ (1)

Formula (1) delineates the Level-1 model, where the intercept π_0i_ signifies the performance of the i-th athlete at age L. The coefficient π_1i_ denotes the instantaneous growth rate of the i-th athlete’s performance at age L, while π_2i_ signifies the acceleration of each growth trajectory.


$$\pi_{pi}=\beta_{p0}+\sum \beta_{pq}X_{qi}+r_{pi}$$ (2)

Formula (2) depicts the Level-2 model, where “p” represents the intercept and two coefficients from the Level-1 model, while “q” symbolizes the independent variables that affect the Level-2 model.

The fixed effects component of the Level-1 model is employed to estimate the average quadratic trend in the relationship between age and performance. The random effects component of the Level-1 model is employed to estimate the distinctive quadratic trend in the advancement of athletic performance for each athlete in relation to age. The independent variables in the Level-2 model typically comprise individual athlete variables (in this study, excellence age), all of which have fixed effects in the model. The examination of the impact of each independent variable in the Level-2 model on the trajectory of athlete performance development is possible through the use of fixed effects.

#### 2.3.2 Model setting

Stata15.5 software was used for statistical analysis. First, establish an unconditional model. Eq (3) is a layer-1 model, which is used to generate a mean quadratic trend in the relationship between an athlete’s age and performance. “Y_ti_” represents the best performance of the “i” athlete per year. The age at time “t” of the“i” athlete is denoted by “a_ti_”, which is calculated as follows: a_ti_ = athlete age_i_− 18 (the age range for athletic level growth of the athletes was 18–32 years old on average in this study). “π_0i_” denotes the athlete’s performance at age 18, “π_1i_” denotes the athlete’s growth rate of performance at age 18, and “π_2i_” is the changing rate in the athlete’s growth rate of performance (acceleration). The layer-1 model’s error term is represented by “e_ti_”.


$${\text{Y}}_{\text{ti}}=\pi_{0\text{i}}+\pi_{1\text{i}}{\text{a}}_{\text{ti}}+\pi_{2\text{i}}{\text{a}}_{\text{ti}}^{2}+{\text{e}}_{\text{ti}}$$ (3)

The layer-2 model is shown in Eq (4). The model uses the performance at age 18 (π_0t_), the growth rate at age 18 (π_1t_), and the acceleration (π_2t_) of the “i” athlete of the layer-1 model as dependent variables, but no layer-2 independent variables are defined. The model gives useful empirical data for selecting the best personal growth equation, as well as a reference for the layer-2 model that can be successfully built.


$$\begin{array}{l} \pi_{\text{0i}}=\beta_{00}+{\text{r}}_{0\text{i}}\;\left(\text{Performance}\;\text{model}\right)\\ \pi_{\text{1i}}=\beta_{10}+{\text{r}}_{1\text{i}}\;\left(\text{Growth}\;\text{rate}\;\text{model}\right)\\ \pi_{\text{2i}}=\beta_{20}+{\text{r}}_{2\text{i}\;}\;(\text{Acceleration}\;\text{model}) \end{array}$$ (4)

By incorporating the excellent age variables in the layer-2 model(as in Eq (5), we could examine the differences in their subsequent performance trajectories between athletes who reached excellence at different ages. Equation

$$\begin{array}{l} \pi_{\text{0i}}=\beta_{00}+\beta_{01}{\text{age}}_{0\text{i}}+\beta_{02}{\text{control}}_{0\text{i}}+{\text{r}}_{0\text{i}}\;\left(\text{Performance}\;\text{model}\right)\\ \pi_{\text{1i}}=\beta_{10}+\beta_{11}{\text{age}}_{1\text{i}}+\beta_{02}{\text{control}}_{1\text{i}}+{\text{r}}_{1\text{i}}\;\left(\text{Growth}\;\text{rate}\;\text{model}\right)\\ \pi_{\text{2i}}=\beta_{20}+\beta_{21}{\text{age}}_{2\text{i}}+\beta_{22}{\text{control}}_{2\text{i}}+{\text{r}}_{2\text{i}}\;(\text{Acceleration}\;\text{model}) \end{array}$$ (5)

Subsequently, utilizing the unconditional model, the values of π_0_, π_1_, and π_2_ can be calculated. Based on the residual of each athlete with respect to these three values, the individual values of π_0i_, π_1i_, and π_2i_ can be determined for each athlete. The peak age of the athlete can be using the formula (x = -π_1_/(2π_2_)). The peak performance and the PIA for each athlete can also be calculated using Eq (3). A general linear regression model (OLS) Eq (6) should be established to examine the relationship between the excellent age and peak age, peak performance, and PIA. Additionally, Eq (7) will be established to investigate the potential for a U-shape between the aforementioned variables. In Eqs (6) and (7), the dependent variables are peak age, peak performance, and PIA, while the independent variable in both cases is excellent age.


$${\text{Page}}_{\text{i}}=\beta_{\text{i}}+\beta_{1}{\text{Eage}}_{\text{i}}+{\text{e}}_{\text{i}}$$ (6)

$${\text{Page}}_{\text{i}}=\beta_{\text{i}}+\beta_{1}{\text{Eage}}_{\text{i}}+\beta_{2}{\text{Eage}}_{\text{i}}^{2}+{\text{e}}_{\text{i}}$$ (7)

## 3. Results

Table 1 shows the descriptive statistics of the original data from this study, categorised by gender and event.

**Table 1:** Descriptive statistics of the original data by gender and event.

	N	Male	Performance/σ	N	Female	Performance/σ
		Age/years			Age/years	
10,000 M	440	25.92±4.797	0.72±0.719	382	27.46±5.332	0.51±0.685
100 M	913	25.9±5.237	0.56±0.562	830	24.93±5.141	0.70±0.728
1500 M	750	25.82±4.892	0.66±0.738	780	25.84±5.127	0.49±0.849
200 M	900	25.55±4.895	0.54±0.59	906	24.64±4.957	0.67±0.81
400 M	678	25.57±4.731	0.47±0.791	857	25.34±4.846	0.55±0.886
5000 M	670	25.92±5.032	0.79±0.815	700	26.39±5.468	0.59±0.771
800 M	631	25.21±4.799	0.62±0.816	756	25.39±5.274	0.43±0.798
High Jump	541	25.61±5.165	0.69±1.015	471	24.8±5.441	0.62±1.047
Long Jump	595	25.91±4.946	0.59±0.79	426	24.89±5.2	0.65±0.886
pole vault	560	26.05±5.192	0.69±0.881	444	24.58±5.14	0.82±1.096
Triple Jump	461	26.43±5.647	0.55±0.849	442	25.63±5.528	0.54±0.948
Sum	7139			6994		

Note: “N” is the total number of competitions in which the athlete has participated. “Performance” is the standardised score.

**Fig 1:**
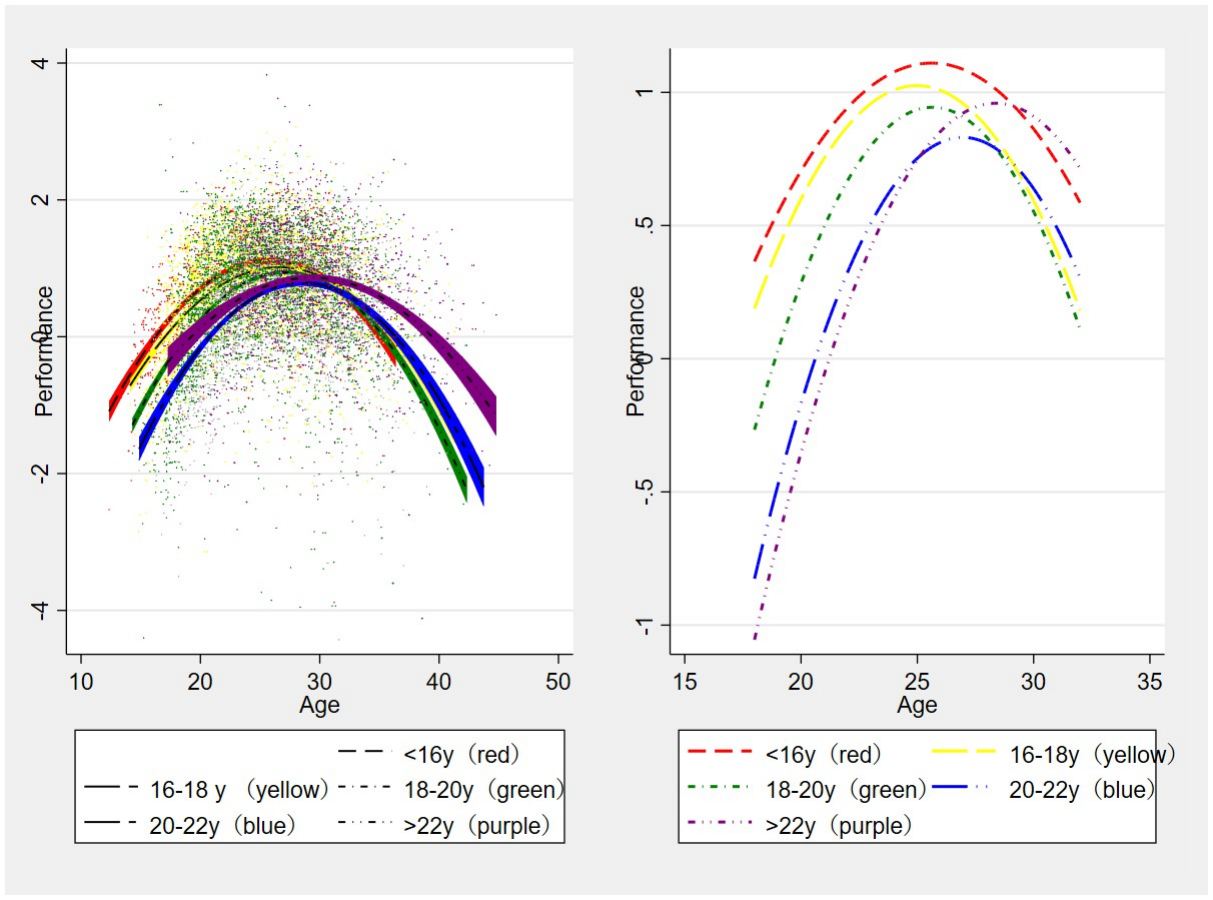
The performance trajectories of the athletes reaching excellence at different ages. The left figure is plotted based on the original data, while the right figure is plotted based on the results of the linear model.

An unconditional model was established (see Table 2). We defined the thresholds for different age groups as follows: <16, 16–18, 18–20, 20–22, and >22 years based on excellent age. Subsequently, excellent age was incorporated into the layer-2 model following the unconditional model. The results are shown in Table 1, indicating a significant difference in the performance trajectory associated with excellent age, as evidenced by the LR test (LR = 329.5; p < 0.001) compared to the model without the excellent age variable. This outcome is visually depicted in Fig 1.

**Table 2:** Model fitting results with age variable at excellent level.

	unconditional model			Model (Excellent age)		
	Performance model	Growth rate model	Acceleration model	Performance model	Growth rate model	Acceleration model
Fixed Effect						
	-0.351*** (0.031)	0.295*** (0.007)	-0.017*** (0.001)			
<16y				0.365*** (0.120)	0.196*** (0.028)	-0.013*** (0.018)
16-18y				-0.177 (0.136)	0.044 (0.032)	-0.004** (0.002)
18-20y				-0.632*** (0.130)	0.121*** (0.031)	-0.008*** (0.002)
20-22y				-1.191*** (0.146)	0.174*** (0.034)	-0.008*** (0.002)
>22y				-1.420*** (0.173)	0.192*** (0.038)	-0.006** (0.002)
Random effect						
performance at age 18	0.904***(0.042)			0.154** (0.006)		
Growth rate at age 18	0.212***(0.008)			0.011** (0.0005)		
Acceleration	0.012***(0.001)			0.685** (0.024)		
Layer 1 error	0.483***(0.033)			0.506** (0.003)		

Note: The performance model, Growth rate model, Acceleration model represents how the independent variables of the stratum-2 model affected the performance at age 18, growth rate at age 18, and acceleration of the stratum-1 model. In the unconditional model, there are no independent variables. In contrast, in Model 1, Excellent Age serves as the independent variable. The group younger than 16 years is the reference category.

Fig 1 illustrates an inverted U-shaped relationship between peak performance and excellent age, while both peak age and PIA appear to increase with increasing excellent age. Nonetheless, statistical testing is necessary to confirm these results. Consequently, regression models (OLS) were constructed with peak performance, peak age, and PIA as dependent variables, and excellent age as the independent variable. The descriptive statistics of key variables in the model are presented in Table 3, and the results are shown in Table 4.

**Table 3:** Descriptive statistics of variables in the model.

	Mean	SD	95%IC
Excellent age	19.53	2.641	19.34~19.72
Peak performance	1.556	0.621	1.512~1.600
Peak age	26.78	7.228	26.28~27.30
PIA	1.280	1.079	1.204~1.356

**Table 4:** The impact of excellent age on peak performance, peak age, and PIA.

	Peak performance		Peak age		PIA	
	Model 1	Model 2	Model 3	Model 4	Model 5	Model 6
Excellent age	-0.014** (0.007)	-0.192*** (0.068)	0.417*** (0.047)	0.484 (0.455)	0.102*** (0.010)	0.628*** (0.095)
Excellent age^2		0.004*** (0.002)		-0.002 (0.011)		0.013*** (0.002)
Gender	Control	Control	Control	Control	Control	Control
Event	Control	Control	Control	Control	Control	Control
R^2^	0.10	0.12	0.04	0.04	0.19	0.21
n	775	775	775	775	775	775

Note: the samples were individually 5 percent shrink-tailed according to peak performance, peak age, and PIA. The dependent variables for Model 1 and Model 2 are Peak performance; for Model 3 and Model 4, they are Peak age; and for Model 5 and Model 6, they are PIA.

Table 4 indicates that (1)when the dependent variable is peak performance, excellent age is significant in both Model 1 and Model 2. Given that Model 2 has a higher R^2^, it can be posited that as excellent age increases, athletes’ peak performance exhibits a "U" relationship. Calculations show that the lowest point was 21.9 years old. (2) When the dependent variable is peak age, the effect of excellent age is only significant in Model 3. Therefore, as excellent age increases, athletes’ peak age gradually rises. (3) In both Model 5 and Model 6, the dependent variable of PIA is significantly influenced by Excellent age. The results of Model 6 indicate an accelerated increase in PIA as Excellent age increases. Combining the results of Model 5 and Model 6, it can be concluded that as Excellent age increases, PIA gradually improves.

Given the more intricate interrelationship between excellent age and peak performance, athletes will be classified into three categories: speed(100 m, 200 m, 400 m,), endurance(800 m, 1 500 m, 5 000 m, 10 000 m,), and fast-power(pole vault, high jump, long jump, and triple jump). This will enable a more detailed examination of the relationship between excellent age and peak performance.

Table 5 reveals that (1) in the Speed category, Excellent age is only significant in Model 1, indicating that as Excellent age increases, peak performance gradually decreases for speed-oriented athletes. (2) In the Endurance category, Excellent age is only significant in Model 4, suggesting that as Excellent age increases, peak performance for endurance athletes initially decreases and then increases. The calculations indicate that the inflection point occurs at 22.0 years old. (3) In the fast-power category, the Excellent age is not significant in both models. This suggests that there is no relationship between Excellent age and peak performance for fast-power athletes.

**Table 5:** The impact of excellent age on peak performance varies across different events.

	Speed		Endurance		Fast-Power	
	Model 1	Model 2	Model 3	Model 4	Model 5	Model 6
Excellent age	-0.215** (0.009)	-0.175 (0.112)	-0.013 (0.010)	-0.248** (0.114)	-0.0001 (0.017)	0.040 (0.237)
Excellent age^2		0.004 (0.003)		0.005** (0.003)		-0.001 (0.006)
Gender	Control	Control	Control	Control	Control	Control
R^2^	0.14	0.14	0.06	0.08	0.06	0.06
n	273	273	306	306	232	232

Note: The samples were individually 5 percent shrink-tailed according to peak performance.

## 4. Discussion

The hypothesis was validated: significant discrepancies were observed in performance development as athletes reached excellence at different ages. The study revealed that as athletes’ age at reaching excellence increases, their peak age rises, and their PIA increases in parallel. The observed increase in peak ages and PIA can be attributed to the enhanced trainability of athletes across both temporal and amplitude dimensions. The primary reasons typically leading athletes to achieve excellence at later ages include (1)weaker athletic talent: Some athletes may inherently possess higher athletic qualities, enabling them to achieve excellence more rapidly. Conversely, others may require a longer training period to reach the same level [21]. 2) Training limitations: Some athletes may have commenced training at a later age or may have been exposed to less scientifically or effectively designed training programs and methodologies in the past. As a result, this delay may have negatively impacted their ability to achieve excellence. [22] Consequently, athletes with lower proficiency at an early stage or slightly weaker athletic talents tend to display higher trainability during adulthood. This observation is consistent with the training response curve proposed by Issurin, Yessis [21], which indicates that as athletes’ proficiency improves, their response to training decreases. Athletes who achieve excellence at a later stage may still be in the initial phase of the training response curve during early adulthood. This phase is characterized by rapid progress in both training and competition, along with an enhanced responsiveness to training stimuli. Athletes who achieve excellence at a later stage may encounter a plateau phase of the learning curve during early adulthood. This phase is characterized by challenges resulting from physical limitations and technical bottlenecks, which may lead to slower progress and a reduction in responsiveness to training stimuli.

The study reveals a U-shaped relationship between excellent age and peak performance. This suggests that athletes achieving excellence either at a earlier or later age tend to perform better at their peak. Conversely, athletes reaching excellence during an intermediate age range may experience lower peak performance in the future. These findings imply that athletes who achieve excellence early or later may possess certain advantages, contributing to better peak performances. This contrasts with previous research, which primarily focused on a wide range of adolescent athletes, including those who would go on to be successful and those who would not [16, 23–26], whereas our study concentrated on athletes who had already succeeded in international competitions.

The study additionally indicated that in endurance events, a U-shaped relationship was observed between excellent age and peak performance, while a significant negative correlation was found in speed events, and no correlation was observed in fast-power events. This variance is attributed to genetic limitations on primary athletic abilities across different sports and disparities in training response.

The findings of the research indicate that between 60% and 70% of maximal oxygen uptake (VO2 max) is indeed determined by genetics [27, 28]. In contrast, with prolonged training, the increase in mitochondrial enzyme activity and muscle capillary density can exceed 100% [29]. Moreover, research has demonstrated that in endurance events, high training volume and previous running experience are among the most crucial factors for enhancing athletes’ running economy [30]. Running economy significantly benefits the performance of endurance athletes. Notably, the running economy tends to gradually improve with the duration of training years [30]. The aforementioned research indicates that one of the primary determinants of endurance events, maximal oxygen uptake, is to some extent constrained by genetics. This suggests that athletes with superior athletic talents are more likely to achieve higher career performances. Nevertheless, factors related to aerobic capacity demonstrate a significant response to training. Moreover, the high trainability of running economy substantially enhances an athlete’s running efficiency. These factors permit athletes with limited athletic talents in endurance events to enhance their performance by improving peripheral adaptations and running economy. Consequently, in such events, as athletes reach older ages of excellence, peak performance demonstrates a "U"-shape.

The study demonstrated that genetic factors account for approximately 70% to 80% of the variation in anaerobic capacity and peak blood lactate levels [28]. Following extensive training, the peak anaerobic power of young athletes may increase by 20% to 30%, while their anaerobic capacity (total power output over 30 seconds) may rise approximately 20% [31]. These studies indicate that one of the primary determinants of speed events, maximal anaerobic endurance, is largely influenced by genetics, and its response to training is relatively low. Consequently, in speed events, athletes with limited athletic talents encounter significant challenges in substantially enhancing their career peak performance through improvements in training adaptations. These factors contribute to the phenomenon where such athletes, as they reach excellence at a later age, experience a lower career peak performance.

The factors that determine maximal anaerobic power in fast-power events are predominantly influenced by genetics. In contrast, fast-power events such as the high jump and pole vault feature significantly more intricate movement patterns compared to speed events. Hollings, Hopkins, and Hume [20] posit that in track events, the same movement patterns recur across multiple cycles. Consequently, the performance in these events is more dependent on the expression of raw strength. In field events such as the high jump and long jump, multiple running action cycles are coordinated with various individual movements to create a series of intricate movement patterns. These events ultimately entail applying explosive force generated by the legs onto the ground to generate a reaction force, necessitating the acquisition and application of skills related to the expression of raw strength. The technical maneuvers inherent to field events necessitate a longer training period [32]. Furthermore, research has demonstrated that the genetic predisposition for coordination abilities is approximately 40% [10]. Georgiades, Klissouras, Baulch, Wang, and Pitsiladis [33] also posit that deliberate practice has a more pronounced impact on enhancing athletes’ performance, particularly in activities that require a high degree of skill but exhibit highly predictable movement patterns. Consequently, the development of athletic skills is minimally influenced by genetics but necessitates long-term training. In summary, in events with higher technical demands, athletes can enhance their competitive performance through skill acquisition and learning, even when their physical capabilities are limited. Therefore, in fast-power events, athletes with limited athletic talents can still improve their performances to some extent by enhancing their skills. These factors contribute to the phenomenon where the peak age and peak performance of such athletes are unrelated.

**Fig 2:**
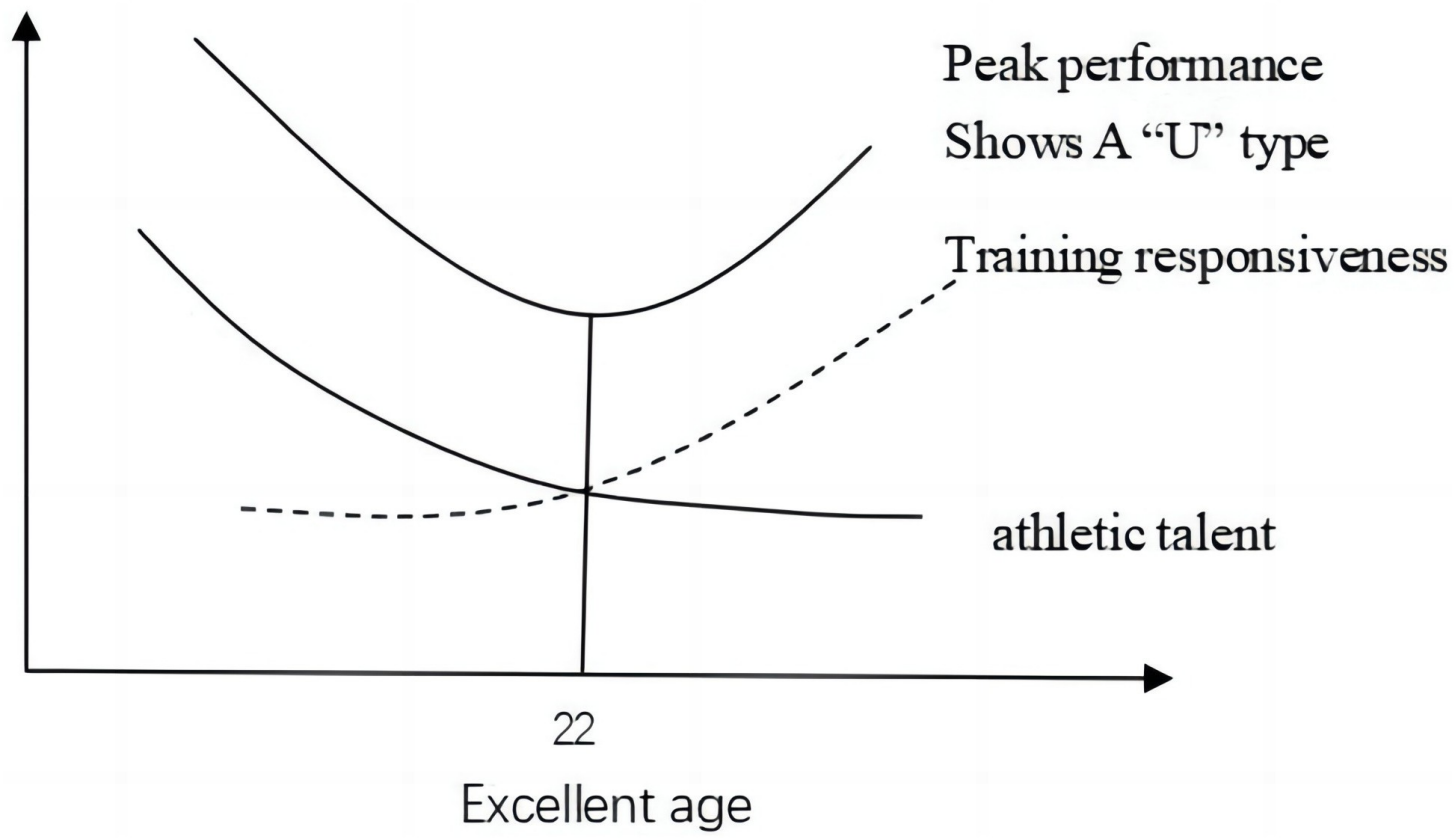
The relationship between athletic ability talent, training adaptability, and the age at which athletes reach excellence.

Based on our analysis, we can infer that athletes who achieve excellence at an earlier age may possess greater athletic talent, while those who achieve excellence at a later age may exhibit stronger training responsiveness (e.g., Fig 2). Given that the lower limit of the U-shaped relationship is at 22 years of age, we propose the following hypothesis: (1) Athletes who achieve excellence before the age of 22 may experience a gradual decline in athletic talent as their excellent age increases, while their training responsiveness may gradually strengthen. However, since the negative impact of declining talent outweighs the positive impact of enhanced training responsiveness, athletes’ peak performance exhibits a declining trend. (2) athletes who achieve excellence after the age of 22, the rate of growth in their training responsiveness accelerates. Despite a decline in athletic talent, the positive impact of enhanced training responsiveness outweighs the negative impact of declining talent. Therefore, as excellent age increases, athletes’ peak performance exhibits an upward trend. These two scenarios collectively manifest as a "U"-shaped relationship, wherein athletes with high athletic talent or high training responsiveness tend to achieve higher peak performances in the future. Of course, this phenomenon may vary depending on the proportion of various motor abilities in different sports.

In summary, athletes possessing exceptional athletic talent or high training responsiveness also bring a range of positive influences to their future performances, including physical condition, training processes, athletic experience, and psychological factors. Athletes who possess high athletic talent and achieve excellence at earlier age may have the following advantages:(1) More Resources: the athletes are likely to attract more sponsorship and support, facilitating better training conditions in the early stages of their careers [16]. (2) More High-Level Competition Experience: the athletes have more time during their careers to continuously improve their skills and performances in higher-level competitions. Research suggests that athletes who engage in high-level competitions early on are more likely to achieve success in their careers [23]. (3) Better Physical Condition: the athletes have a earlier peak age, indicating that they maintain relatively good physical condition during their peak years, experiencing fewer injuries and fatigue. (4) Greater Motivation: the athletes may have more confidence and motivation, driving them to actively pursue success.

Athletes with high training responsiveness who achieve excellence at a later age may have the following advantages: (1) Enhanced Athletic Performance Efficiency: athletes with a higher peak age have typically undergone more extensive training periods and accumulated greater training experience. This is advantageous for improving their athletic economy, which plays a vital role in enhancing performance in specific sports [34] (2) Stronger Mental Resilience: the athletes may possess greater mental toughness, enabling them to better cope with setbacks and pressure. (3) Expanded Expertise: the athletes are likely to amass greater experience and knowledge, enabling them to make wiser decisions as they progress into the mid-career phase.

## 5. Practical application

We assume that the long-term development pattern of athletes can be customized based on their early performance. Combining the research findings from this study(Refer to Fig 1), it can be observed that:

(1) Athletes who attain excellence before the age of 16 years have an absolute advantage (best performance in all age groups) between the ages of 24 and 28 years and have a relative advantage (best performance in the same age group) until the age of 29 years. (2) The critical period for most athletes who reach excellence at the age of 16–20 years to win major competitions is between the ages of 24 and 26 years. However, extending the sporting life of these athletes after the age of 28 years is laborious and of little avail. (3) Athletes who attain excellence between the ages of 18 and 20 tend to have shorter and lower peak performances. However, if they peak at the right time, they still have the opportunity to achieve victory in competitions between the ages of 25 and 26. (4) Athletes who attain excellence after the age of 22 years have a relative advantage after the age of 29 years, and there is still significant value in extending their sports life at the age of 30 years. These athletes can continue to increase their training volume and intensity until the age of 29 years, which enhances their athletic performances by causing the body to adapt to extremely high loads. However, as achieving good results in competition is the main factor influencing athletes’ continued participation in sports, these athletes may be eliminated early owing to their lower performances before the age of 20 years. In conclusion, Table 6 summarizes the features of athletes’ performance development in each group.

**Table 6:** Advantages and disadvantages of the athletes reaching excellence at different ages.

	<16 years	16–18 years	18–20 years	20–22 years	>22 years
Absolute advantage	24–28 years	-	-	-	-
Relative Advantage	Before age 29	-	-	-	After age 29
Peak age	25.7	25.0	25.7	27.0	28.9
Features	Showing high performance for a long time	Extending sporting life after age 28 is fruitless.	Age 25–26 is a critical period for winning athletic glory.	Avoid being perceived as a late bloomer owing to rapid growth in performance.	There is great value in extending sporting life after age 29.

## 6. Conclusion

In this study, we delved into the relationship between the age at which athletes achieve excellence and their future performance development. Firstly, we found that athletes who achieve excellence at a earlier or later age tend to have better peak performance. Nonetheless, we underscored the notable distinctions between these two categories of athletes, particularly regarding the age at which they attain peak performance and performance improvement during adulthood. This observation may reflect the characteristic trend where, as athletes achieve excellence at later ages, their athletic talent gradually declines while their training responsiveness steadily improves. Finally, we propose that early athletic performance can better predict their future development trends, thereby providing strong support and guidance for personalized long-term training plans. In summary, the age at which athletes attain excellence can lead to distinct advantages in their future performances and development, highlighting the significance of harnessing these disparities to help each athlete reach their maximum potential.

## 7. Limitations and future study

Several limitations are associated with this study. First, the athletes in this study competed for the first time at the IAAF level from 1993 to 2008. Athletes who showed an inflection point in their performance trajectories after 12 years, when they participated in IAAF for the first time, were excluded from the study sample. Therefore, the study results may be affected by sample selectivity bias. Second, athletes’ performance trajectories may not be consistent around the peak age, so setting the same acceleration may have been an oversimplification with a quadratic growth model. Third, many other factors influence the development of athletes’ performance, such as cultural, economic, family, and training science factors. Some factors are likely to be correlated with the variables above. Owing to potential variable omission, there may have been some interference in the relationship between the factors and the trajectories.

In this study, we chose the average performance of athletes at the age of 20 as the excellent performance. However, this choice was somewhat arbitrary. Additionally, we acknowledge that the level of excellence directly affects the number of athletes included in the sample. When the level of excellence is set too high, some athletes may not meet the criteria and may be excluded from the sample, potentially impacting the study’s results. Therefore, further exploration of the relationship between excellent age and the performance trajectory under different levels of excellence will be an important direction for future research. This will enhance our understanding of how achieving excellence at various ages can have diverse effects on athletes’ performance, thereby facilitating more in-depth research in this field. Furthermore, the relationship between athletic talent, training responsiveness, and the excellent age proposed in this study remains speculative in nature and requires further empirical validation.

## Supporting information

S1 Data (XLS)
